# Therapeutic properties of *Helicobacter pylori*-derived vacuolating cytotoxin A in an animal model of chronic allergic airway disease

**DOI:** 10.1186/s12931-023-02484-5

**Published:** 2023-07-06

**Authors:** Jonas Raspe, Mona S. Schmitz, Kimberly Barbet, Georgia C. Caso, Timothy L. Cover, Anne Müller, Christian Taube, Sebastian Reuter

**Affiliations:** 1 Department of Pulmonary Medicine, Experimental Pneumology, University Hospital Essen – Ruhrlandklinik, Tueschener Weg 40, Essen, 45239 Germany; 2 Vanderbilt University Medical Center, Nashville, TN USA; 3 Veterans Affairs Tennessee Valley Healthcare System Nashville, Nashville, TN USA; 4 Institute of Molecular Cancer Research, University of Zurich, Zurich, Switzerland

**Keywords:** Asthma, *Helicobacter pylori*, Vacuolating cytotoxin A, Chronic allergic airway disease model, Regulatory T cells, Tissue residential memory T cells

## Abstract

**Background:**

It has previously been shown that the *Helicobacter pylori* (*H. pylori*)-derived molecule vacuolating cytotoxin A (VacA) could be suitable for the treatment of allergic airway disease. The therapeutic activity of the protein, which acts through modulation of dendritic cells (DC) and regulatory T cells (Tregs), was demonstrated in murine short-term acute models. The aim of this study is to further evaluate the therapeutic potential of VacA by determining the effectiveness of different application routes and the suitability of the protein for treating the chronic phase of allergic airway disease.

**Methods:**

VacA was administered by the intraperitoneal (i.p.), oral (p.o.) or intratracheal (i.t.) routes, and long-term therapeutic effectiveness, allergic airway disease hallmarks, and immune phenotype were analyzed in murine models of acute and chronic allergic airway disease.

**Results:**

Administration of VacA via the i.p., p.o or i.t. routes was associated with a reduction in airway inflammation. The i.p. route showed the most consistent effect in reducing airway inflammation and i.p. treatment with VacA was the only treatment that significantly reduced mucus cell hyperplasia. In a murine model of chronic allergic airway disease, both short- and long-term treatment with VacA showed a therapeutic effect, with a reduction in a variety of asthma hallmarks, including bronchoalveolar lavage eosinophilia, lung inflammation and goblet cell metaplasia. Short-term treatment was associated with induction of Tregs, while repetitive long-term administration of VacA influenced immunological memory in the lung.

**Conclusions:**

In addition to showing therapeutic efficacy in short-term models, treatment with VacA also appeared to be effective in suppressing inflammation in a chronic airway disease model. The observation that treatment was effective after administration via several different routes highlights the potential of VacA as a therapeutic agent with different routes of administration in humans.

**Supplementary Information:**

The online version contains supplementary material available at 10.1186/s12931-023-02484-5.

## Background

It is estimated that 300 million people worldwide are affected by asthma [1], making it one of the most common non-communicable chronic diseases. Asthma is characterized by a multitude of different disease phenotypes and endotypes [2]. Although the underlying pathophysiological mechanisms of asthma are now well described, existing treatment options are purely symptomatic rather than curative. The microbiome and its interactions with the immune system are promising starting points in the search for new therapies [3].

Surprisingly, the gastric bacterium *Helicobacter pylori* (which is associated with gastritis and gastric ulcer) has emerged as a promising candidate for treating asthma. Epidemiological studies [4, 5] and investigations in mouse models [6] have shown that neonatal infection with *H. pylori* protects against the development of asthma in later life. Studies with knock-out *H. pylori* mutants showed that the protein vacuolating cytotoxin A (VacA) plays a substantial role in mediating the protective effects of *H. pylori* in asthma [7]. It was subsequently shown that both prophylactic and therapeutic intraperitoneal (i.p.) treatment with purified VacA can suppress the development of an allergic respiratory disease in mice [8, 9].

In both prophylactic and therapeutic models, the suppressive effect of VacA was associated with immunomodulation of dendritic cells (DC) and the induction of regulatory T cells (Tregs). Furthermore, we were able to obtain initial indications that VacA might be translationally effective in humans [9]. Treatment of human DC with VacA induced the secretion of anti-inflammatory cytokine interleukin (IL)-10 and induced Tregs in autologous DC/T cell co-culture.

This study was designed to shed light on two central questions concerning the therapeutic effectiveness of VacA. Firstly, which route of administration is the most effective and would be best suited for the treatment of allergic respiratory disease? Secondly, is VacA effective in an animal model of chronic allergy respiratory disease, and are there differences between therapeutic short-term and long-term treatment approaches with respect to attenuation of asthma hallmarks and immune-modulating effects? Investigating these questions should provide further insights into the therapeutic potential of VacA for the treatment of asthma.

## Methods

### Animals

The animals used in the experiment were SPF C57BL/6JRj mice purchased from Janiver Labs [Le Genest-Saint-Isle, France] and housed under SPF conditions at the Laboratory Animal Facility of the University Hospital Essen. The mice were all female and aged between 8 and 12 weeks at the beginning of the experiments.

### VacA

The VacA used in these experiments was an active strep-tagged oligomeric s1m1 type VacA derived from modified forms of the *H. pylori* strain 60,190. VacA was isolated and purified as described previously [10].

### House dust mite (HDM)

A lyophilized extract derived from whole bodies of *Dermathophagoides pteronyssinus* (*D. pteronyssinus*) was obtained from Greer Laboratories [#XPB82D3A2.5] and used for all experiments. *D. pteronyssinus* represents the most common mite species in Germany and Europe, and is therefore the main source of HDM allergens for European individuals with atopy [11]. The lyophilized cake was reconstituted with phosphate-buffered saline (PBS) to a concentration of 1 mg/mL adjusted to the protein content. The Der p 1 concentration was 13 µg/mL.

### Acute allergic airway disease model

**Fig. 1:**
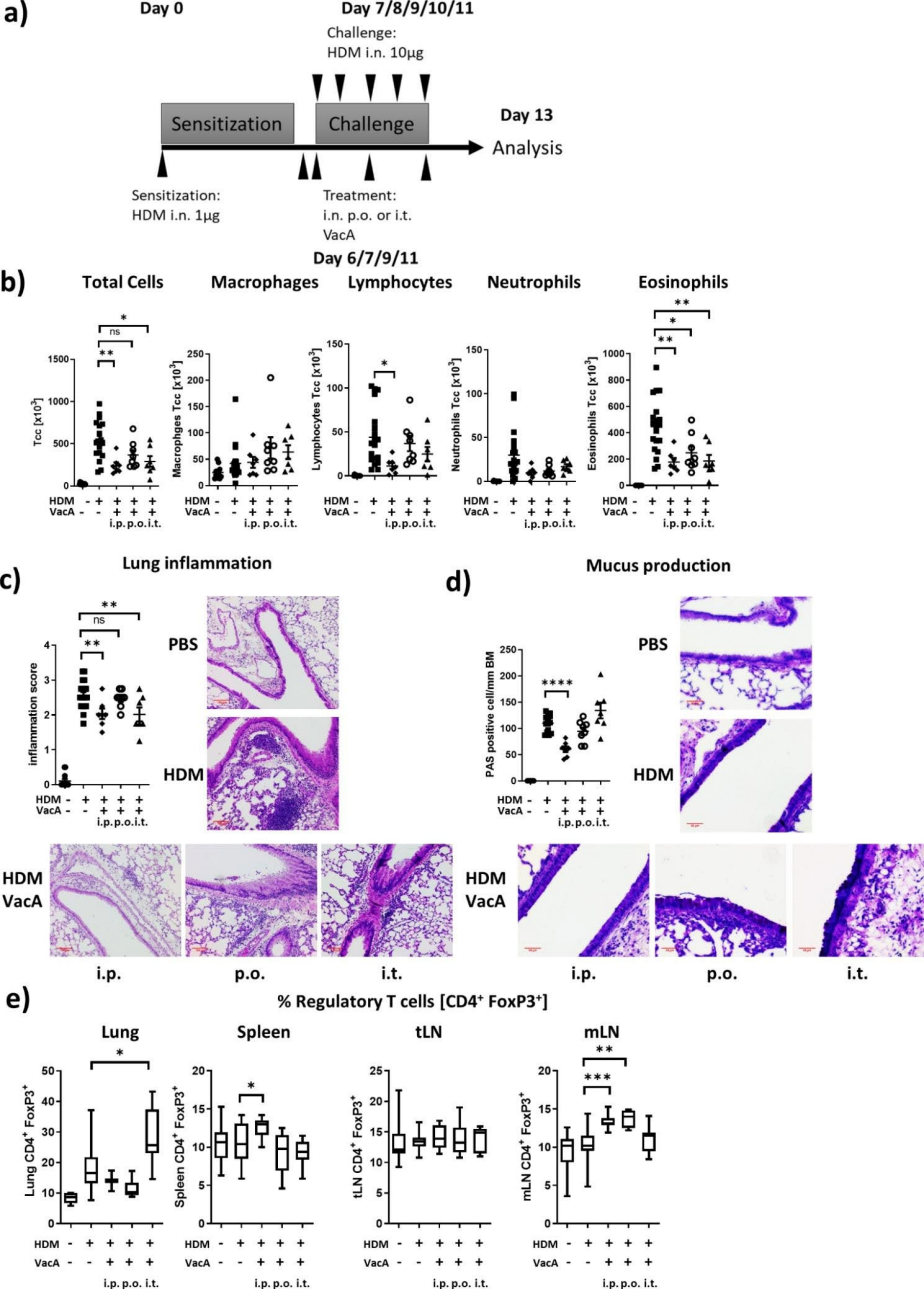
Application route dependent effects of VacA on the asthma phenotypes. **a)** On day 0 all animals were sensitized intranasally (i.n.) with 1 µg house dust mite (HDM); on day 7–11, the positive control and the three different VacA-treated groups were challenged i.n. with 10 µg HDM. The negative control group was challenged with phosphate-buffered saline (PBS). On days 6, 7, 9 and 11, animals were treated 20 µg VacA given via the intraperitoneal (i.p.), oral (p.o.) or intratracheal (i.t.) route. **b)** Cellular composition of bronchoalveolar lavage (BAL): total cell count (Tcc), macrophages, lymphocytes, neutrophils and eosinophils. **c)** Lung tissue inflammation: representative sections of each indicated group are shown (x100); scatter plot of inflammation score. **d)** Mucus-producing cells: pictures show representative sections from each group (x200); scatter plot of the number of mucus producing cells per mm of basement membrane. **e)** VacA treatment increases proportions of Tregs. Tregs were characterized by the expression of FoxP3^+^ CD25^+^ cells in the CD3^+^ CD4^+^ cell population; box plots show the percentage in lung, spleen, tracheal lymph node (tLN) and mesenteric lymph node (mLN); results from six independent experiments n = 6–15 per group. n.s. not significant; *p < 0.05; **p < 0.01; ***p < 0.001; ****p < 0.0001

Acute allergic airway disease was induced as previously described [12]. On day 0, 1 µg of HDM protein was dissolved in 50 µL of PBS and administered intranasally (i.n.) to isoflurane-anesthetized C57BL/6JRj mice to trigger sensitization. To induce allergic airway disease, the anesthetized mice were challenged i.n. with 10 µg HDM dissolved in 50 µL PBS daily from day 7–11. 20 µg VacA dissolved in PBS was given via the i.p., per oral (p.o.) or intratracheal (i.t.) route on days 6, 7, 9 and 11; i.t. treatments were administered to animals that had been anesthetized with a mixture of ketamine and xylazine [both Serumwerk Bernburg]. Final analysis was performed on day 13 (Fig. 1A).

### Activation model

**Fig. 2:**
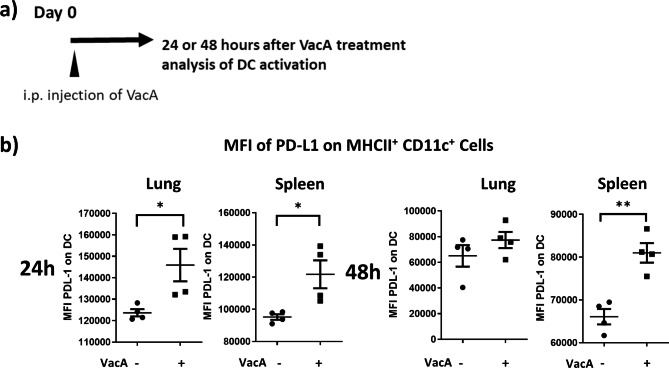
VacA treatment affects PD-L1 expression on MHCII^+^CD11c^+^cells. **a)** 20 µg intraperitoneal (i.p.) VacA was injected into C57BL/6JRj mice and PD-L1 on dendritic cells (DC) was analyzed after 24 or 48 h. **b)** Scatter plots show geomean fluorescence intensity (MFI) of PD-L1 on MHCII^+^ CD11c^+^ cells after 24 or 48 h in lung and spleen; results from n = 4 per group. *p < 0.05; **p < 0.01

C57BL/6JRj mice were treated i.p. with VacA 20 µg dissolved in PBS, and analyzed after 24 and 48 h (Fig. 2A).

### Chronic allergic airway disease model

C57BL/6JRj mice were sensitized and challenged as described in the acute allergic airway disease model. Subsequently, mice received either 50 µL PBS or 1 µg HDM dissolved in 50 µL PBS twice a week for 10 weeks. Allergen and PBS were applied i.t. to avoid oral tolerance towards HDM. Animals were anesthetized with ketamine/xylazine and then the PBS or HDM solution was carefully injected directly into the airways with a pipette.

**Fig. 3:**
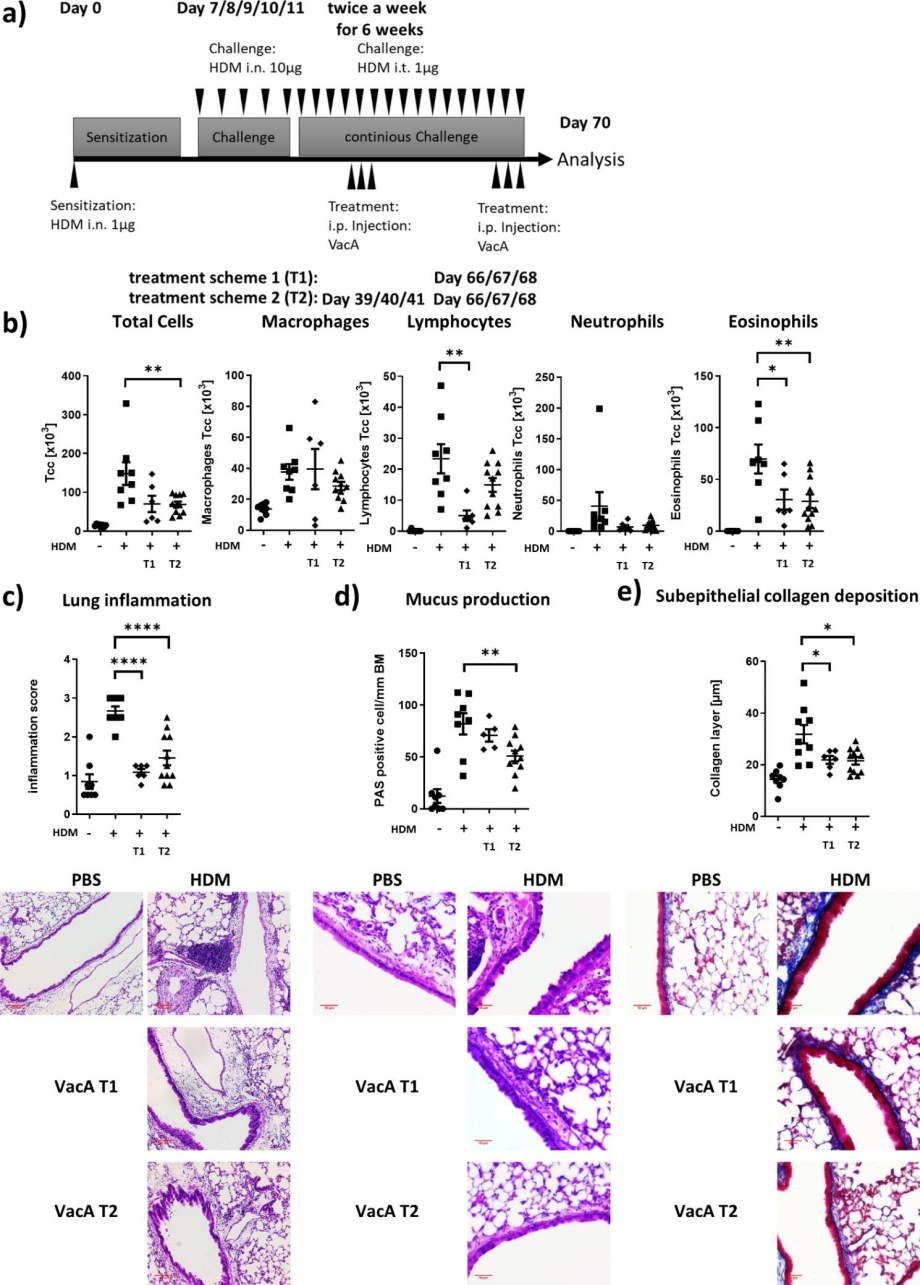
Effects of VacA treatment in a model of chronic allergic airway disease. **a)** Mice were sensitized on day 0 with 1 µg of house dust mite (HDM) given intranasally (i.n.). Mice were challenged with 10 µg HDM on days 7, 8, 9, 10 and 11. Subsequently, the positive control and the VacA-treated groups were challenged with 1 µg HDM intratracheally (i.t.) twice a week for 6 weeks. During the chronic challenge phase, two treatment schemes were applied: short-term (T1) = 20 µg intraperitoneal (i.p.) VacA at days 66, 67 and 68, and long-term (T2) = 20 µg VacA i.p. at days 39, 40, and 41 plus days 66, 67 and 68. **b)** Cellular composition of bronchoalveolar lavage (BAL): total cell count (Tcc), macrophages, lymphocytes, neutrophils and eosinophils. **c)** Lung tissue inflammation: representative sections of each indicated group are depicted (x100); scatter plot depicts inflammation score. **d)** Mucus-producing cells: pictures show representative sections from each group (x200); scatter plot depicts the number of mucus producing cells per mm of basement membrane. **e)** Subepithelial collagen deposition: pictures show representative sections from each group (x200); scatter plot shows averaged subepithelial collagen layer thickness in µm for each group; results from two independent experiments n = 5–9 per group. *p < 0.05; **p < 0.01; ****p < 0.0001

Two different treatment regimens with VacA were performed. For short-term treatment (T1), VacA 20 µg dissolved in 100 µL PBS was administered i.p. on three consecutive days in the last week of the experiment (week 10, days 66/67/68). For long-term treatment (T2), VacA 20 µg dissolved in 100 µL PBS was administered i.p. on three consecutive days in the middle and the last week of the experiment (week 6, days 39/40/41 and week 10, days 66/67/68). Analyses were performed 24 h after the last VacA treatment (Fig. 3A).

### Assessment of asthma hallmarks

To assess the impact of VacA on the asthma phenotype, the composition of the bronchoalveolar lavage (BAL) fluid, infiltration of immune cells into the lung tissue, the number of mucus-producing cells, and subepithelial collagen deposition were analyzed, and the immune phenotype was assessed.

### Analysis of BAL

Lungs were flushed with 1 mL ice-cold PBS through an i.t. tube and total cell counts were determined. To determine differential cell counts, cytocentrifuged preparations of BAL were stained with Hemacolor-Set (Merck) and at least 200 cells were counted and differentiated into macrophages, lymphocytes, neutrophils and eosinophils based on histological properties. The total cell count for each cell type was calculated based on relative cell counts and the initially determined total cell number.

### Lung histology

Tissue sections for histological analyses were prepared as previously described [13]. In short, after the removal of the left lung lobe, the right lung lobe was inflated with Histofix (Roth) and then transferred to the fixative. After embedding in paraffin, lung sections with a thickness of 2.5 μm were cut with a microtome. Sections were stained with hematoxylin/eosin (HE) to assess the degree of inflammation, or with combined Periodic Acid Schiff (PAS)/HE staining to identify mucus-producing goblet cells. Tissue sections were stained with Masson Trichrome staining to determine subepithelial collagen deposition.

The degree of lung inflammation was assessed by observers who were unaware of experimental groups, who scored five randomly selected areas on a scale from 0 (no visible infiltrate around airway vessels and parenchyma) to 4 (several layers thick cellular infiltrates on nearly all visible vessels and airways). A more detailed description of the individual scores has been reported previously [13]. Mucus-producing cells were quantified per millimeter of basal membrane on three different representative airways on PAS-stained slides. Subepithelial collagen deposition was determined by measuring the thickness of collagen deposition on five different positions in at least three airways. The mean thickness was first calculated for each airway and then across the three airways.

### Assessment of the immune phenotype

The immune phenotype was analyzed in four different organs: lung, spleen, lung draining tracheal lymph node (tLN), and mesenteric lymph nodes (mLN) using flow cytometry. First, single cell suspensions were produced as briefly described below. The lung was flushed with PBS via the right ventricle to eliminate venous blood before preparation. The left lung lobe was eviscerated, minced with a scalpel and transferred into 50 mL reaction tubes. A collagenase digestion was performed by adding collagenase type I (0.5 mg/mL; catalog no. C9891, Sigma) and incubating in a shaking water bath at 37 °C for 45 min. Subsequently, cells were passed through a cannula (20G 0.9 mm x 40 mm) and transferred through a 70 μm cell strainer into a new tube. Cell counts were determined after erythrocyte lysis (KHCO_3_ 20mM/L [Roth], NH_4_Cl 310mM/L [Roth], EDTA 200µM/L [Sigma] in aqua dest.). The spleen was extracted and pushed through a 70 μm cell strainer, erythrocytes were lysed, and cell counts were determined. Lymph nodes were extracted and ground between the roughened ends of two glass microscope slides, washed with Hanks’ balanced salt solution, and filtered with a 70 μm cell strainer, then cell counts were determined. All single cell solutions were adjusted to 1 × 10^7^ cells/mL with IMDM (PAN Biotech; w L-Glutamine; 25mM HEPES, 3,024 g/L NaHCO_3_) containing 10% fetal calf serum (FCS; PAA Laboratories), 1% Pen/Strep (Gibco; 10.000 U/mL penicillin; 10.000 µg/mL streptomycin) and stored on ice until further processing.

### Flow cytometry

Measurements were performed on the CYTOFLEX LX platform of Beckman Coulter. The generated data were analyzed using the FlowJo Software Version 10.6.1 which also was used to generate the cytometry graphics.

Single-cell solutions containing 5 × 10^5^ cells were used for each flow cytometric staining. Staining was performed in a 96-well plate (TC Plate 96 Well Suspension, R [Sarsted]). Live- dead staining was performed using Zombie UV in a dilution of 1:1000 (Zombie UV™ Fixable Viability Kit [BioLegend]) to exclude dead cells. Subsequently, binding cells were blocked with 0.5 µL of Fc receptor-blocking antibodies (TruStain FcX™ [anti mouse CD16/32 Isotype Rat igG2a, λ clone: 93 Biolegend]) to reduce non-specific antibody binding.

To identify DC and T cells, samples were incubated for at least 15 min with the appropriate antibody mixtures. After surface staining, a FoxP3 intracellular staining was performed to mark Tregs, as recommended by the manufacturer.

#### General analysis strategy

at the beginning of each analysis, doublets and debris were excluded based on their size and shifting properties in FSC-A and FSC-H. After exclusion of dead cells (Zombie UV positive cells), CD45-positive cells were selected and then auto-fluorescent cells were excluded based on their signal in an empty channel not occupied by antibodies.

#### Strategy to identify Tregs

within the non-auto-fluorescent cells, T helper cells were identified based on the expression of CD3 and CD4. Within these, Tregs were selected as FoxP3-positive cells. Details of the antibodies used and a gating example are provided in Supplementary Tables 1 and Supplementary Fig. 2.

#### Strategy to identify MHCII expression on DC

within non-fluorescent hematopoietic cells, CD19^+^ B cells were excluded and CD11c/MHCII-positive cells were selected. After exclusion of CD3^+^ cells in this population, the geomean fluorescence intensity (MFI) of MHCII on DC was determined. The antibodies used are listed in Supplementary Tables 2 and an example gating is shown in Supplementary Fig. 3.

#### Strategy to identify memory T cells

within the group of hematopoietic cells, T cells were identified as CD3-positive cells; T helper cells were distinguished from CD8-positive cytotoxic T cells. Naïve cells were characterized as CD62L^+^CD44– cells, central memory cells as CD62L^+^CD44^+^ cells, effector memory cells as CD62L^–^CD44^+^ cells, and tissue residential memory cells as CD69^+^CD103^+^ cells. The antibodies used are detailed in Supplementary Tables 3 and an example gating strategy is shown in Supplementary Fig. 4.

### Statistical analysis

An Anderson Darling test was performed to determine whether data followed a Gaussian approximation, and then the appropriate statistical test (unpaired Student t-test or Mann-Whitney U test) was used to compare between groups. Statistical significance was defined as a p-value of ≤ 0.05.

## Results

**VacA administration by different routes attenuates allergic respiratory disease**.

An acute model of allergic airway disease in mice was used to test whether VacA is therapeutically effective via different routes of administration and to identify the most effective route (Fig. 1A). Administration of VacA via i.p., p.o., and i.t. routes reduced the number of eosinophilic granulocytes compared with the untreated positive control (Fig. 1B). Although none of the treatment regimens reduced the number of neutrophils and macrophages, i.p. application of VacA significantly reduced lymphocytes in the BAL. Both i.p. and i.t. application of VacA also significantly reduced total cells in the BAL.

Administration of VacA via the i.p. route reduced the inflammation in the lung tissue (Fig. 1C) and decreased the number of mucus-producing goblet cells in the airways (Fig. 1D); lung inflammation was also reduced after i.t. (but not p.o.) administration of VacA (Fig. 1C), but and neither of these routes of administration altered the number of mucus producing cells in the airways (Fig. 1D).

Flow cytometric analysis of FoxP3^+^/CD25^+^ Tregs showed an organ-specific increase of Tregs depending on the VacA route of administration. After i.t. treatment there was a significant increase in Tregs in the lungs, after i.p. treatment there was an increase of Tregs in the spleen and the mLN, and after p.o. administration there was an induction of Tregs in the mLN compared with the positive control (Fig. 1E).

### VacA treatment induces PD-L1 on DC in vivo

In our previous work we could observe an induction of PD-L1 on the surface of human DC after stimulation with VacA in vitro. To investigate whether VacA can also modulate the immunosuppressive surface ligand on DC in vivo, animals were treated i.p. with VacA, and then PD-L1 expression on DC in different organs was examined 24 or 48 h later (Fig. 2A). A significant upregulation of PD-L1 was observed in the lung- and spleen-derived DC as early as 24 h after treatment with VacA. This was still detectable in spleen DC at 48 h after treatment (Fig. 2B).

### VacA suppresses the inflammatory respiratory phenotype in a chronic model of allergic airway disease

The therapeutic effectiveness of VacA in the chronic phase of allergic airway disease was determined in animals sensitized to HDM and continuously challenged with the allergen over 10 weeks. Both short- and long-term treatment with VacA were associated with improvement in the inflammatory phenotype (Fig. 3A). Short-term treatment significantly reduced lymphocytes and eosinophils in the BAL, and decreased lung inflammation and sub-epithelial collagen deposition compared with the positive control. Long-term treatment with VacA reduced eosinophilia in the BAL, which was accompanied by a lower lung inflammation score, lower numbers of mucus-secreting goblet cells in the airways and less sub epithelial collagen deposition (Fig. 3B-E).

### Effects of VacA on the immune phenotype vary with short- and long-term treatment

**Fig. 4:**
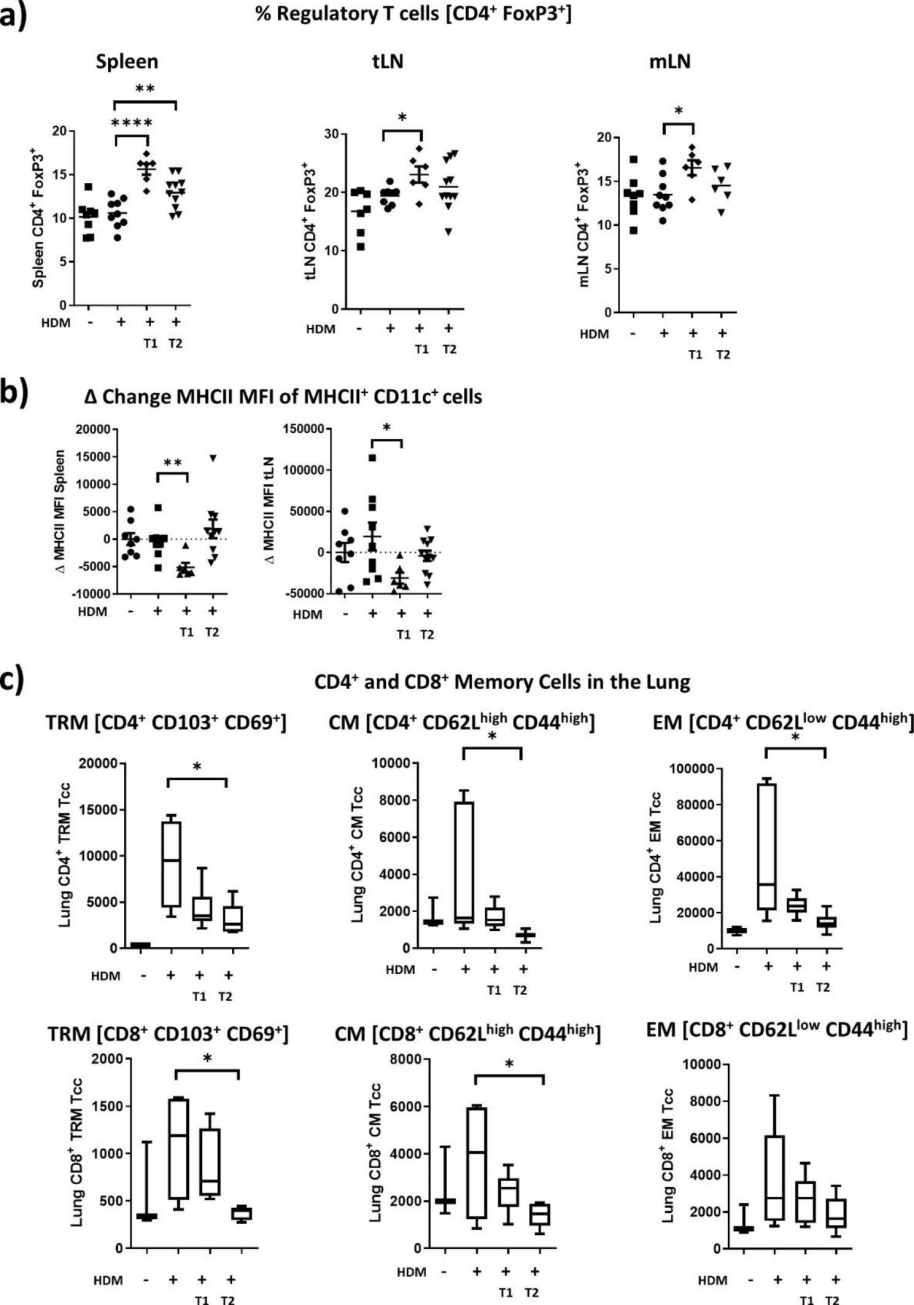
Effects of VacA treatment on the immune phenotype in a chronic allergic airway disease model. **a)** VacA treatment increases proportions of Tregs. Tregs were characterized by the expression of FoxP3^+^ CD25^+^ cells in the CD3^+^ CD4^+^ cell population; scatter plots show the percentage in lung, spleen, tracheal lymph node (tLN) and mesenteric lymph node (mLN). **b)** Scatter plot showing change in geomean fluorescence intensity (MFI) of MHCII on MHCII^+^ CD11c^+^ cells versus negative control group. **c)** Box plot of tissue residential memory T cells (CD103^+^ CD69^+^), central memory T cells (CD62L^high^ CD44^high^) and effector memory T cells (CD62L^low^ CD44^high^) in the CD3^+^ CD4^+^ and CD3^+^ CD8^+^ cell populations; results from two independent experiments n = 5–9 per group. *p < 0.05; **p < 0.01; ****p < 0.0001

To analyze the effect of the different VacA treatment approaches on the immune phenotype, Tregs, MHCII expression on DC and induction of memory T cells were analyzed in lung, spleen, mLN and tLN in the chronic model (Fig. 4). Short-term treatment was associated with an increased proportion of Tregs in spleen, mLN and tLN. After long-term treatment, an increased proportion of Tregs could only be detected in the spleen compared to the positive control (Fig. 4A). Previously, we could demonstrate that DC in the draining lymph nodes of VacA treated animals exhibited reduced expression of MHCII on the surface in an acute model of allergic airway disease.

Similarly, also in the chronic model short-term (but not long-term) treatment with VacA decreased expression of MHCII on DC (CD11c^+^/MHCII^+^) in the lung draining lymph node and the spleen (Fig. 4B).

Development of an immunological memory represents a central step in adaptive immunity, and is important for the induction of recall responses against pathogens, but is a disadvantage if these responses are directed against a harmless allergen [14]. Treatment with VacA, especially long-term treatment, attenuated the development of an immunological memory in the lung. CD4^+^ and CD8^+^ tissue residential memory (TRM- CD103^+^/CD69^+^) were detectable in reduced numbers in the lung. In addition, numbers of CD4^+^ and CD8^+^ central memory cells (CM - CD62L^high^/CD44^high^) and effector memory cells (EM – CD62L^low^/CD44^high^) were lower after long-term treatment with VacA (Fig. 4C).

To evaluate any possible side effects of VacA treatment, animals were observed throughout the experiments and their body weight was recorded; no visible signs of distress or differences in body weight were seen compared with the positive control group (Supplementary Fig. 1A). In addition, no cytotoxic effects were detected in VacA-treated animals based on flow cytometry analyses (Supplementary Fig. 1B).

## Discussion

The results of this study confirm and substantiate the suitability of VacA as therapeutic treatment option for asthma. These findings extend previous data showing that the i.p. administration of VacA can suppress the development and progression of an allergic respiratory disease [9]. New insights from the current study include the documentation of the therapeutic effects of VacA after administration via several different routes, and demonstration that VacA can downregulate inflammatory responses in a chronic model of allergic airway disease, without any signs of adverse effects after repeated applications.

The inhaled, oral, subcutaneous or intravenous routes are the typical ways patients with asthma take their medications. Of these, oral intake of tablets or inhalation using an inhaler have the best acceptability and compliance. In the present study, we compared the effectiveness of equivalent concentrations of VacA administered via i.p. injection, p.o. delivery via gavage or i.t. delivery of droplets into the lung in a murine model of acute allergic airway disease. All three of these were effective, although there were differences in the magnitude of the effects of VacA given via the different routes. All were associated with a significant reduction in the eosinophil count in the BAL, while i.p. and i.t. administration also significantly reduced the total cell count, and i.p. also reduced lymphocytes. In terms of the histological analysis of the inflammatory phenotype, i.p. and i.t. administration of VacA significantly reduced the infiltration of cells into lung tissue, while i.p. treatment also reduced mucus-producing cells in the airways.

Similar to previous studies, all routes of VacA administration in the current study were able to induce Tregs. Tregs, and modulation of DC towards a tolerogenic phenotype, are key mechanisms of action underlying the therapeutic effectiveness of VacA [15]. In this study, we showed that the induction of Tregs occurs primarily at the site of VacA administration, with significant induction in the lung after i.t. administration, in the spleen and mesenteric lymph nodes after i.p. administration, and in mesenteric lymph nodes after p.o. administration.

Overall, the findings of this study show that VacA can have a therapeutic effect via different application routes. Further, our results indicate that i.p. application represents the most effective and consistent therapy approach with the current formulation of the molecule. However, a different formulation and dosage of VacA could potentially increase its effectiveness via treatment routes like inhalation or oral administration, which are the preferred treatment options for humans with higher patient compliance; this remains to be determined in future studies.

We propose that modulation of DCs represents a central mechanism in VacA-mediated immune suppression. Previous studies have shown that *H. pylori*, and VacA, can induce mouse and human DC with a tolerogenic phenotype [9, 16]. These DC are capable of secreting anti-inflammatory mediators such as IL-10, and also show increased expression of immune receptors with inhibitory motifs on their surface.

Recently, we showed that VacA induced the expression of PD-L1 on the surface of human DCs in vitro [9]. In general, upregulation of the PD-L1/PD-1 pathway is important for the development, maintenance and function of induced Tregs [17]. PD-L1 is strongly associated with the modification of the T-cell immune response [18] and appears to be important for the generation of regulatory T-cells [19]. The role of the PD-L1/PD-1 pathway in allergic diseases and asthma is controversial. PD-L1 expression is associated with reduced proliferation of allergen-specific human CD4^+^ T cells [20]. A human PD-1 agonist was shown to alleviate neutrophilic asthma [21] whereas new-onset asthma has been reported as a side effect of cancer treatment with the PD-1 inhibitor nivolumab [22]. It is assumed that the PD-1/PD-L1 axis is involved in regulating the severity of allergic asthma by targeting Th17 cell activity [23]. However, PD-L2 (but not PD-L1) has been discussed to have a beneficial effect in asthma [24]. Interestingly, the induction of Treg-inducing tolerogenic PD-L1-expressing human DC is also described as the immune suppressing mechanism of allergoid-mannan, a newly described vaccine for allergen-specific immunotherapy [25]. In the current study, i.p. administration of VacA increased expression of PD-L1 on DCs in the lungs and spleen. By increasing the expression of PD-L1 on DC, VacA can directly modulate the communication between DC and T cells and promote the generation of anti-inflammatory T cells.

To date, all experiments investigating the therapeutic efficacy of VacA have been carried out in acute models, in which the effect of the molecule during the onset of the disease or shortly thereafter has been analyzed. Asthma is a chronic lung disease in which patients experience recurrent inflammatory reactions in the lungs, resulting in remodeling of the airways, which in turn contributes to disease progression. This study used a HDM-specific mouse model of chronic allergic airway disease to determine whether VacA was also effective in the chronic phase of the disease and evaluate the safety of repeated doses of VacA. After an initial sensitization and a one-week challenge period, the allergen was administered i.t. at low doses to mice over 6 weeks. Two different VacA treatment regimens were examined: short-term treatment at the end of the experiment and long-term treatment given in the middle and the end of the experiment. Both treatment regimens significantly reduced eosinophilia in the BAL, lung tissue infiltration and subepithelial fibrosis. However, there were also some differences between effects of the two regimens. Short-term treatment significantly reduced the number of lymphocytes in the BAL, while long-term treatment significantly reduced the total number of cells in the BAL and the mucus-producing cells. These differences in the regulation of the inflammatory phenotype of the allergic airway disease were accompanied with variances in the immune phenotype of the animals.

Similar to observations in the acute and therapeutic model, short-term treatment with VacA in the chronic model was associated with visible induction of Tregs in the lung draining lymph node, spleen and mLN; this induction was less pronounced in animals receiving long-term VacA treatment. In addition to the induction of Tregs, we also observed a modulation of DC after short-term treatment. Similar to what we saw in the acute model of allergic airway disease, we detected reduced expression of MHCII on DC in lung and spleen, but only during short-term (not long-term) treatment. The current findings also showed that VacA can suppress the inflammatory phenotype in the chronic model. Here, the beneficial effects of short-term treatment were accompanied by induction of Tregs and modulation of DC. The anti-inflammatory cytokine IL-10, which has an important role in the induction and maintenance of T cell tolerance, can reduce MHCII expression on DC, and the reduction of MHCII on the surface of DC then leads to T cell unresponsiveness or anergy [26]. These tolerogenic DC can also upregulate the activity of Tregs that subsequently suppress the underlying inflammation [27].

An interesting finding of the current study was that long-term treatment with VacA seemed to have an impact on the development of immunological memory. VacA reduced the number of both CD4^+^ and CD8^+^ tissue resident, central and effector memory T cells in the lung. Studies have shown that CD4 tissue resident memory T cells (TRM) are responsible for the existence and recurrence of asthma [14], and that CD8 TRM also play an important role in the recruitment of immune cells to the lungs, especially in chronic cases [28]. These TRM can persist in the lung for a long time and maintain the local “allergic memory”, which is associated with the induction of asthma exacerbations upon repeated exposure to an allergen [14]. This makes them an interesting target for the treatment of allergic asthma.

Overall, the observations in the current study underline the therapeutic capacity of VacA in asthma because both short- and long-term treatment in a chronic disease model were able to alleviate the asthma phenotype. Long-term treatment seems to have an impact on the development of local immunological memory. A reduction of memory against the allergen in the lung could be an interesting new approach to treat asthma. No obvious adverse effects were detected in the current study, but impact on the immune modulation on other immune responses, for example infections, needs to be evaluated.

## Conclusions

The *H. pylori*-derived molecule VacA appears to be a promising candidate for the treatment of allergic diseases such as allergic asthma when given via a variety of administration routes. In addition to demonstrated activity in acute and acute/therapeutic models of allergic respiratory disease, the present data showed that treatment with VacA can also effectively suppress inflammatory reactions in chronic allergic airway disease models. Overall, VacA appears to be an interesting future therapeutic option that can counter-regulate diseases that are characterized by an excessive immune response (such as asthma) by inducing immunosuppressive mechanisms, thus minimizing disease severity and potentially contributing to long-term remission.

## Electronic supplementary material

Below is the link to the electronic supplementary material.

Supplementary Material 1

## Data Availability

The datasets used and/or analyzed during the current study are available from the corresponding author on reasonable request.

## List of abbreviations

BAL: bronchoalveolar lavage
CM: central memory T cells
*D. pteronyssinus*: *Dermathophagoides pteronyssinus*
DC: dendritic cell
EM: effector memory T cells
*H. pylori*: *Helicobacter pylori*
HDM: house dust mite
i.n.: intranasal
i.p.: intraperitoneal
i.t.: intratracheal
mLN: mesenteric lymph node
PBS: phosphate buffered saline
PD1: programmed cell death protein-1
PD-L1: programmed cell death protein-ligand 1
p.o.: peroral
T1: short-term treatment regimen
T2: long-term treatment regimen
tLN: tracheal lymph node
Treg: regulatory T cell
TRM: tissue residential memory T cells
VacA: vacuolating cytotoxin A
