# Supplementary material for: Therapeutic properties of Helicobacter pylori-derived vacuolating cytotoxin A in an animal model of chronic allergic airway disease
Source: Respir Res. 2023 Jul 6;24:178. doi: 10.1186/s12931-023-02484-5 (PMC10324189; doi:10.1186/s12931-023-02484-5)
Supplement: Supplementary file 1 — Supplementary Material 1 [file 12931_2023_2484_MOESM1_ESM.docx]

**Supplementary material**

**Supplementary Figure 1: No evidence of cytotoxic effects during treatment with VacA.**

a) Scatter plots show the proportion (%) of dead cells in different organs at the end of treatment in the chronic allergic airway disease model; cells were stained with Zombie UV.

b) Change in body weight [g] of the mice (C57BL/6j) during treatment with VacA in the chronic allergic airway disease model (body weight increases over time).

DM, house dust mite; mLN, mesenteric lymph node; PBS, phosphate-buffered saline; Tcc, total cell count; tLN, tracheal lymph node; T1, short-term VacA treatment; T2, long-term VacA treatment.


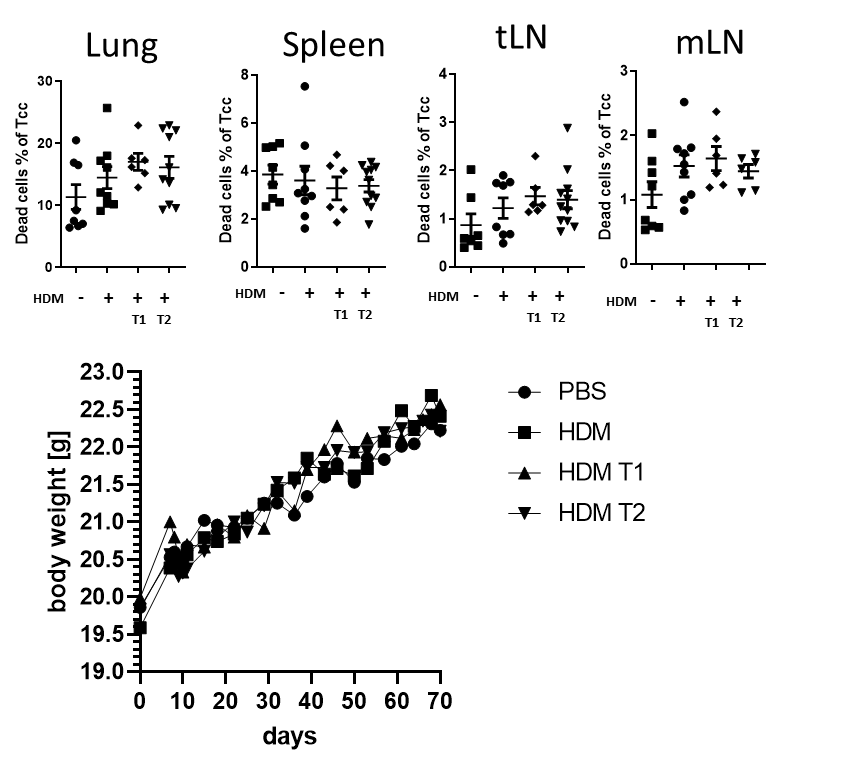


**Supplementary Figure 2: Gating strategy for regulatory T-cells.**

First doublets were excluded then a live/dead staining was performed. CD45^+^ cells were gated and auto-fluorescent cells were excluded. Regulatory T cells (Treg) were identified as CD3^+^CD4^+^FoxP3^+^ cells.

**
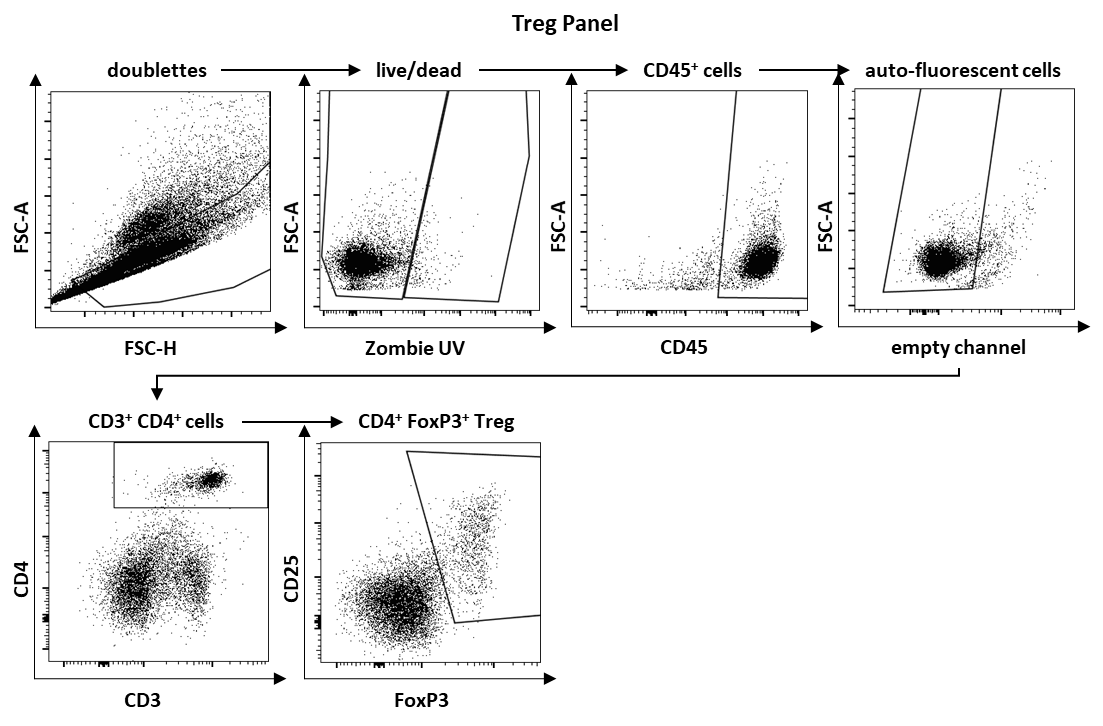
**

**Supplementary Figure 3: Gating strategy for dendritic cells and mean fluorescence intensity (MFI) of MHCII or PD-L1.**

First doublets were excluded then a live/dead staining was performed. CD45^+^ cells were gated and CD19^+^ cells were excluded. CD11c^+^ MHCII^+^ cells were identified and after exclusion of CD3^+^ cells the MFI of MHCII or PD-L1 was determined.


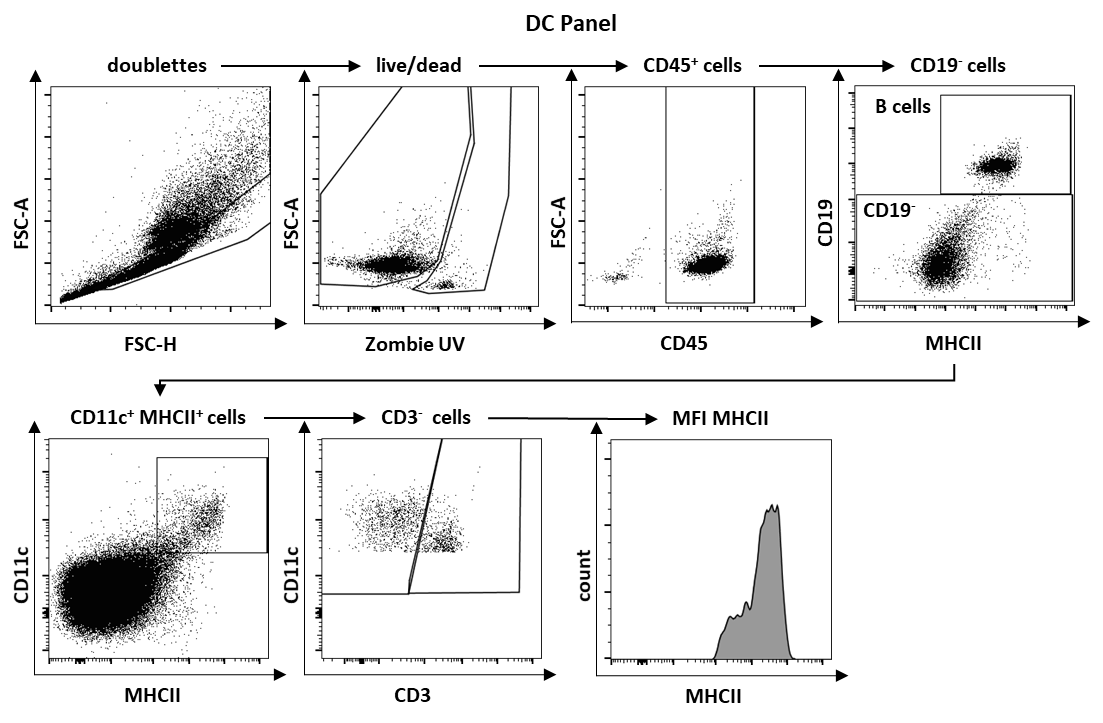


**Supplementary Figure 4: Gating strategy for tissue residential memory T cells (TRM).**

First doublets were excluded then a live/dead staining was performed. CD45^+^ cells were gated and lymphocytes were identified after gating of CD3^+^cells, CD4^+^ and CD8^+^ cells were separated and on each population the memory gating was performed.


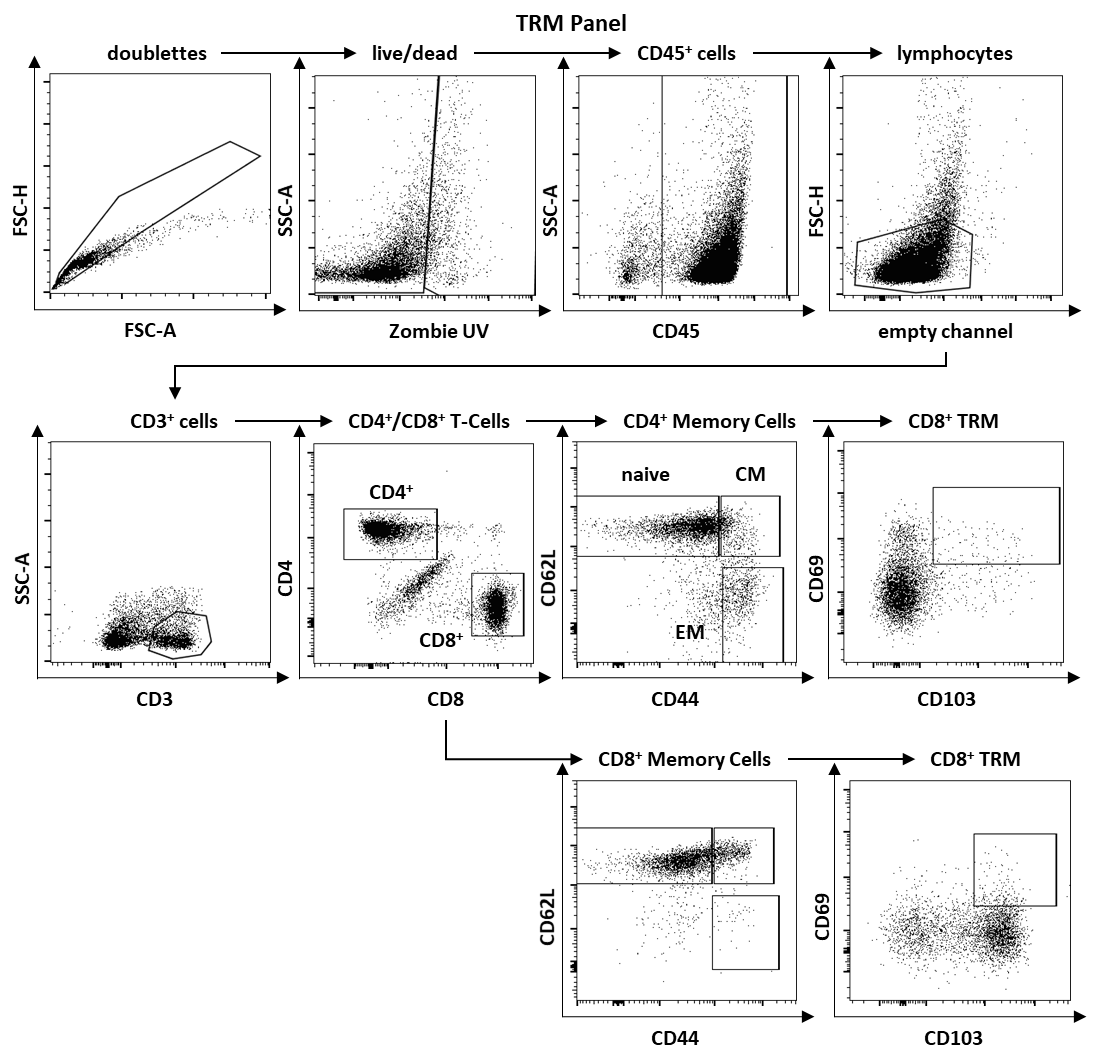


**Supplementary Table 1: Antibody list – regulatory T cells (Treg)**

| **Panel** | **Murine Treg Analysis** | | | | |
| --- | --- | --- | --- | --- | --- |
| **Fluochrome** | **Specificity** | **Clone** | **Reactivity** | **Antibody Host Species** | **Manufacturer** |
| Alexa Fluor 700 | CD45 | 30F-11 | Mouse | rat | Biolegend |
| PerCP-Cy™5.5 | CD3 | 145-2C11 | mouse | rat | Biolegend |
| PE/Cyanine7 | CD4 | 53-6.7 | mouse | rat | Biolegend |
| Pe | CD25 | PC61 | mouse | rat | BD Pharmingens |
| Brilliant Violet 650™ | CD25 | PC61 | mouse | rat | Biolegend |
| APC | FoxP3 | FJK-16s | mouse | rat | Biolegend |

**Supplementary Table 2: Antibody list – MHCII expression on dendritic cells (DC)**

| **Panel** | **Murine DC** | | | | |
| --- | --- | --- | --- | --- | --- |
| **Fluochrome** | **Specificity** | **Clone** | **Reactivity** | **Antibody Host Species** | **Manufacturer** |
| Alexa Fluor 700 | CD45 | 30F-11 | mouse | rat | Biolegend |
| PE/Dazzle™ 594 | CD19 | 6D5 | mouse | rat | Biolegend |
| PE/Cyanine 7 | CD11c | N418 | mouse | armenian hamster | Biolegend |
| FITC | MHCII | M5/114.15.2 | mouse | rat | Biolegend |
| APC/Cyanine 7 | CD3 | 145-2C11 | mouse | armenian hamster | Biolegend |

**Supplementary Table 3: Antibody list – memory T cells**

| **Panel** | **Murine memory T cells** | | | | |
| --- | --- | --- | --- | --- | --- |
| **Fluochrome** | **Specificity** | **Clone** | **Reactivity** | **Antibody Host Species** | **Manufacturer** |
| Alexa Fluor 700 | CD45 | 30F-11 | mouse | rat | Biolegend |
| PerCP-Cy™5.5 | CD3 | 145-2C11 | mouse | rat | Biolegend |
| PE/Cyanine7 | CD4 | 53-6.7 | mouse | rat | Biolegend |
| Brilliant Violet 605™ | CD62L | MEL-14 | mouse | rat | Biolegend |
| Brilliant Violet 785™ | CD44 | IM7 | mouse | rat | Biolegend |
| APC/Cyanine 7 | CD69 | H1.2F3 | mouse | armenian hamster | Biolegend |
| PE | CD103 | 2E7 | mouse | armenian hamster | Biolegend |
| Brilliant Violet 510™ | CD8 | 53-6.7 | mouse | rat | Biolegend |

**Supplementary Table 4: Antibody list – PD-L1 expression on dendritic cell**

| **Panel** | **Murine PD-L1** | | | | |
| --- | --- | --- | --- | --- | --- |
| **Fluochrome** | **Specificity** | **Clone** | **Reactivity** | **Antibody Host Species** | **Manufacturer** |
| Alexa Fluor 700 | CD45 | 30F-11 | mouse | rat | Biolegend |
| PE/Cyanine 7 | CD11c | N418 | mouse | armenian hamster | Biolegend |
| FITC | MHCII | M5/114.15.2 | mouse | rat | Biolegend |
| PE/Dazzle™ 594 | PDL-1 | 10F.9G2 | mouse | rat | Biolegend |
